# Late-onset of pulmonary embolism following hospitalization for COVID-19 despite thromboprophylaxis: a report of two cases

**DOI:** 10.11604/pamj.2021.38.226.28503

**Published:** 2021-03-01

**Authors:** Falmata Laouan Brem, Zakariae Missaoui, Mohammed Arghal, Hammam Rasras, Narjisse Aichouni, Imane Skiker, Noha El Ouafi, Bazid Zakaria

**Affiliations:** 1 Department of Cardiology, Mohammed VI University Hospital, Faculty of Medicine and Pharmacy of Oujda, Mohammed First University, Oujda, Morocco,; 2 Department of Radiology, Mohammed VI University Hospital, Faculty of Medicine and Pharmacy of Oujda, Mohammed First University, Oujda, Morocco,; 3 Epidemiological Laboratory of Clinical Research and Public Health, Faculty of Medicine and Pharmacy of Oujda, Mohammed First University, Oujda, Morocco

**Keywords:** Post-discharge, COVID-19, coagulopathy, pulmonary embolism, case report

## Abstract

The global pandemic caused by the SARS-CoV-2 has resulted in an increased incidence of venous thromboembolism among hospitalized COVID-19-patients, especially those who required intensive care, despite thromboprophylaxis. This has resulted in the use of higher doses of thromboprophylaxis or therapeutic anticoagulation therapy even in the absence of thrombotic events. However, after their hospital discharge, authors and current guidelines are not unanimous about extended anticoagulant therapy in patients with COVID-19. Here, we report two pulmonary embolism cases following hospitalization for COVID-19, despite intermediate doses of thromboprophylaxis. These rare cases suggest that there may be a residual thrombotic risk following hospitalization for COVID-19 and highlight questions about extended prophylactic-anticoagulation therapy after hospital discharge of patients with COVID-19.

## Introduction

Current data reported a high pooled incidence of pulmonary embolism (PE) among hospitalized patients with COVID-19, especially critical ill, despite thromboprophylaxis [1]. This has resulted in the use of higher doses of thromboprophylaxis or therapeutic anticoagulation therapy even in the absence of thrombotic events [2]. However, little is known about post-discharge VTE incidence among patients with COVID-19. Here, we report two post-discharge pulmonary embolism cases in patients with COVID-19 who were discharged under intermediate doses of prophylactic anticoagulation therapy, suggesting that there may be residual thrombotic risk in discharged COVID-19-patients.

## Patient and observation

A retrospective review of two pulmonary embolism cases that occurred after the hospital discharge of patients with COVID-19 disease. They were seen at the University Hospital Center of Oujda between December 12^th^, 2020 and January 16^th^, 2021. We performed COVID-19 tests by RT-PCR on nasopharyngeal swabs. The timeline, clinical characteristics and outcomes of patients are summarized in Table 1.

**Table 1:** timeline

Time	Events
CASE 1	
12 December 2020	Symptoms: dry cough, asthenia, fever, headache, and anosmia
17 December 2020	Hospitalization for the first time for the SARS-CoV-2 infection (RT-PCR for the SARS-CoV-2 was positive). Computed tomography (CT) scan confirmed the SARS-CoV-2 pneumonia. Patient underwent treatment with ceftriaxone, Azithromycin and enoxaparin.
04 January 2021	Discharge with Enoxaparin 6000UI/24h
8 January 2020	Rehospitalization for worsening dyspnea. CTPA showed COVID-19 pneumonia related features and pulmonary embolism.
12 January 2020	Hypovolemic chock then deceased
CASE 2	
14 December 2020	First symptoms: dry cough fever, asthenia and myalgia
18 December 2020	Hospitalization for the first time for the SARS-CoV-2 infection (RT-PCR for the SARS-CoV-2 was positive). Computed tomography (CT) scan confirmed the SARS-CoV-2 pneumonia. Patient underwent treatment with ceftriaxone, Azithromycin and enoxaparin.
28 December 2020	Discharge with Enoxaparin 6000UI/24h
11 January 2021	Rehospitalization for worsening dyspnea ; CTPA showed COVID-19 pneumonia related features and pulmonary embolism.
16 January 2021	Discharge on Rivaroxaban

### Patient 1

**Figure 1:**
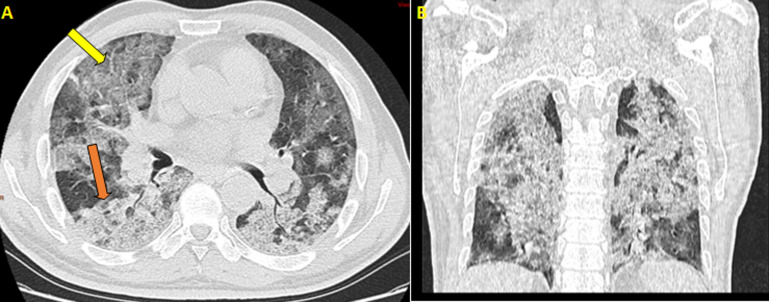
the first thoracic CT scan in an axial (A); and coronal (B) lung window showed ground-glass-opacities associated with crazy paving (yellow arrow) as well as the start of consolidation (orange arrow); the percentage of lung involvement is approximately 75%

**First hospitalization:** a 66-year-old man with diabetes mellitus was admitted to the emergency department (ED) with polypnea (21 cycles/min) associated with fever, cough, headache and anosmia that began five days before. He was stable hemodynamically, oxygen saturation in the room air at 82% (Early warning Score (EWS) at 7). The physical examination was without abnormalities. COVID-19 RT PCR (reverse transcription-polymerase chain reaction) performed on a nasopharyngeal swab showed a positive result. A computed tomography (CT) scan on the first admission showed signs of COVID-19 (Figure 1). Laboratory findings showed a lymphopenia at 460/mm^3^, with elevated inflammatory markers: C-reactive protein (CRP) at 239mg/L and D-dimer level (640μg/L). The patient underwent treatments with low molecular weight heparin (LMWH), enoxaparin 4000UI/24h, ceftriaxone and azithromycin. After 19 days, his condition improved and the patient was discharged with prophylactic-anticoagulation therapy (6000UI/24h).

**Figure 2:**
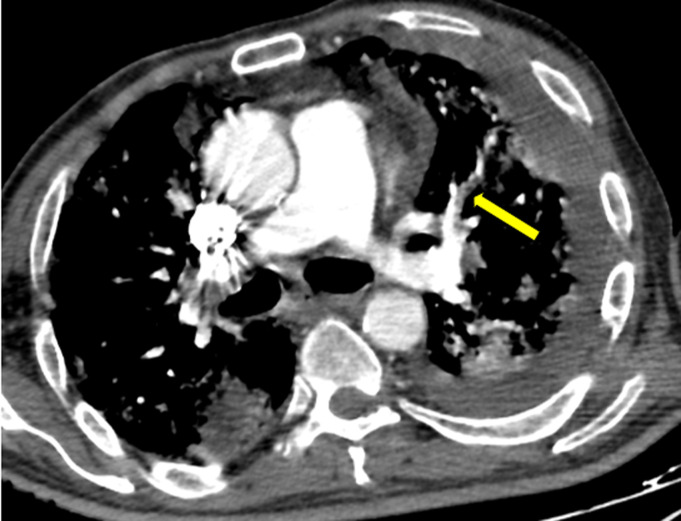
CTPA in an axial window showing an acute pulmonary embolism of the upper lobar branch of the left pulmonary artery (yellow arrow), associated with a left pleural effusion

**Figure 3:**
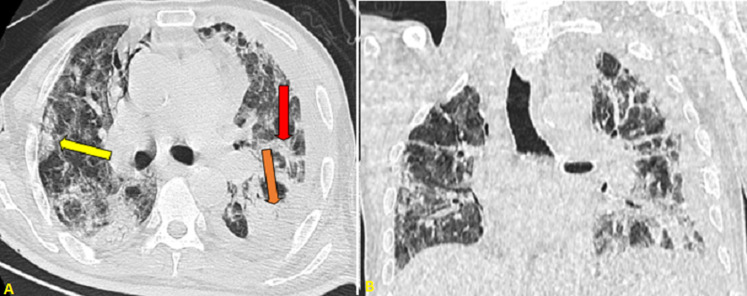
thoracic CT scan in an axial (A); and coronal (B) window objectifying ground glass opacities associated with crazy paving (yellow arrow) and fibrotic bands (red arrow), as well as consolidation in lower lobes (orange arrow)

**Readmission:** four days after discharge, he deteriorated with tiredness and increased breathing (oxygen saturation at 79% at room air). Laboratory findings showed a high level of D-dimer level was (17 000μg/L). A CTPA was performed, which showed a filling defect in the right pulmonary artery (Figure 2) with almost the same degree of lung damage (Figure 3). He underwent a therapeutic dose of anticoagulant therapy with low-molecular-weight heparin. Two days after his admission, the patient presented a hypovolemic shock managed with vasopressor drugs and venous filling. Unfortunately, the patient deceased the fourth day of his second admission.

### Patient 2

**Figure 4:**
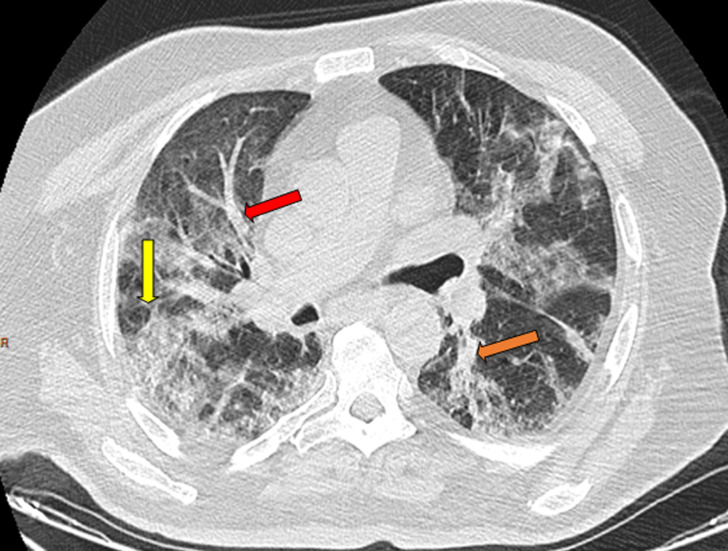
the first thoracic CT scan in an axial lung window showing widespread bilateral ground glass opacities associated with crazy paving (yellow arrow), vascular dilatation (red arrow) as well as consolidation (orange arrow)

**First hospitalization:** a 66-year-old male with no previous medical history was admitted to the emergency department with polypnea (22 cycles/min) associated with a fever, dry cough, myalgia and asthenia that had begun four (4) days before. He was stable hemodynamically, oxygen saturation at 85% in the room air (Early warning score (EWS) at 5). The physical examination was without abnormalities. COVID-19 RT PCR (reverse transcription-polymerase chain reaction) performed on a nasopharyngeal swab showed a positive result. A computed tomography (CT) scan on the first admission showed signs of COVID-19 pneumonia (Figure 4). Laboratory findings showed lymphopenia at 720/mm^3^, with increased values of C-reactive protein (CRP) and D-dimer (1240μg/L, RR < 500μg/L). The patient underwent treatments with low molecular weight heparin (LMWH), enoxaparin 4000UI once a day, ceftriaxone and azithromycin. After ten days, his condition improved, and the patient was discharged with a prophylactic-anticoagulation therapy (6000UI/24h).

**Figure 5:**
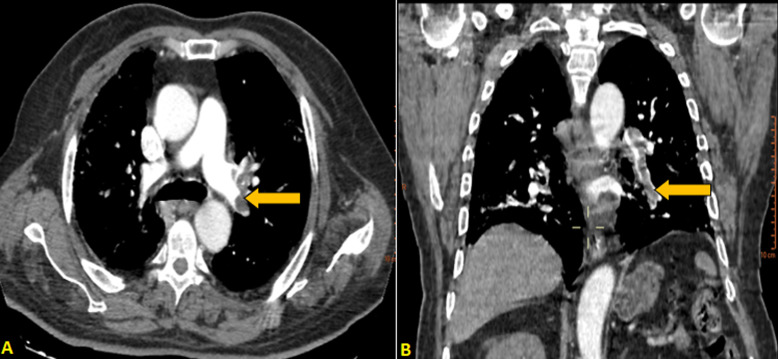
CTPA in axial (A); and coronal (B) windows showing pulmonary embolism of the left pulmonary artery extended to the lower lobar and segmental branches (yellow arrow)

**Figure 6:**
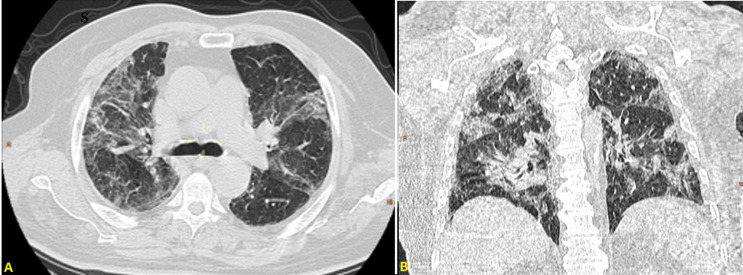
thoracic CT scan in an axial (A); and coronal (B) window showing 60% of CT abnormalities related to for peer review COVID-19 disease

**Readmission:** fourteen days after discharge, he deteriorated with tiredness and increased breathing (oxygen saturation at 80% at room air). Laboratory findings showed a high level of D-dimer at 11400μg/L. A CT pulmonary angiogram (CTPA) was performed, which showed a filling defect in the left pulmonary artery extended to the lower lobar and segmental branches (Figure 5) with 60% of CT abnormalities related to COVID-19 disease (Figure 6). He underwent a therapeutic dose of anticoagulant therapy with low-molecular-weight heparin. The patient was discharged after four days on rivaroxaban and followed-up by his cardiologist.

## Discussion

Indeed, prophylactic-anticoagulant therapy is recommended for all hospitalized COVID-19-patients [3] and has been reported to reduce mortality [4]. Furthermore, the use of higher doses of prophylactic or therapeutic anticoagulation therapy instead of lower standard doses is associated with a lower risk of death and a lower pooled incidence of venous thromboembolism events [2]. However, after hospital discharge, the use of thromboprophylaxis is still a debate. Little is known about venous thromboembolism (VTE) incidence after the hospital discharge of patients with COVID-19. Recently, several studies were conducted to assess the cumulative incidence of post-discharge VTE among patients with COVID-19. They reported a low incidence of post-discharge VTE at 0.48% to 0.7% [5,6] and suggested that hospitalization for COVID-19 disease does not really elevate the risk of post-discharge VTE compared to other acute medical illness. Therefore, extended prophylactic anticoagulant therapy in discharged COVID-19 patients is not routinely needed.

Early data of patients with COVID-19 reported a low rate at 0.6% of 30-day venous thromboembolism and a similar rate at 0.7% of major bleeding [7] Moores LK *et al*. [8] suggested, given that there is almost the same risk of bleeding in patients with and without COVID-19 and similar charge associated with both venous thromboembolism and major bleeding, prolonged prophylactic anticoagulant therapy would provide a benefit, only if the risk of venous thromboembolism at 42- days- post-discharge, is greater than 1.8%. Based on the high risk of VTE, described as patients with an established IMPROVE VTE cut-off score at 4 and D-dimer, (> 2 times the upper limit of normal (ULN)), [9] some authors recommend approximately 14 days at least and up to 30 days after hospital discharge of patients with COVID-19 at high), with either LMWH or the direct oral anticoagulants (rivaroxaban or betrixaban) [10]. We report two post-discharge pulmonary embolism cases in patients with COVID-19 who were discharged under intermediate doses of prophylactic anticoagulation therapy, suggesting that there may be a residual thrombotic risk in discharged COVID-19-patients. However, there have been controversial recommendations on the need for post-discharge thromboprophylaxis from expert guidelines. The American College of Cardiology (ACC) recommends extending prophylactic anticoagulation therapy in patients infected by the SARS-CoV-2 with other risk factors for VTE and high D-dimer levels (two times the ULN or more) at the time of discharge [11] The Italian Society on Thrombosis and Hemostasis (SISET) recommends 7-14 days of post-discharge prophylactic anticoagulant therapy in selected patients with a high risk of venous thromboembolism (obesity, limited mobility, previous VTE, active cancer) [12].

**Informed consent:** written informed consent was obtained from the patient(s) to publish anonymized information in this article.

## Conclusion

In the light of these cases, current guidelines and given the lack of information on the duration of the hypercoagulability state in patients with COVID-19, we suggest that it may be reasonable to extend post-discharge thromboprophylaxis in selected patients at high risk of VTE (reduced mobility, long hospital stay, ICU admission, a previous history of VTE) with either LMWH or direct oral anticoagulants (rivaroxaban or betrixaban). The decision should be made according to the risk-benefit ratio.
